# The crucial role of potassium ion channels in diabetes mellitus and its complications: A review

**DOI:** 10.1080/19336950.2025.2531949

**Published:** 2025-07-12

**Authors:** Xiangdong Yang, Yan Yang

**Affiliations:** a Experimental Medicine Center, The Affiliated Hospital of Southwest Medical University, Luzhou, China; b Key Lab of Medical Electrophysiology of Ministry of Education and Medical Electrophysiological Key Lab of Sichuan Province, Collaborative Innovation Center for Prevention and Treatment of Cardiovascular Disease, Institute of Cardiovascular Research, Southwest Medical University, Luzhou, China

**Keywords:** Diabetes, potassium ion channels, myocardial injury, vascular complications

## Abstract

Potassium ion channel (K^+^ channel) is a crucial transmembrane protein found on cell membranes that plays a pivotal role in regulating various physiological processes such as cell membrane potential, action potential formation, and cellular excitability. Diabetes, a chronic metabolic disorder characterized by elevated blood glucose levels, can cause abnormal changes in the sensitivity and functioning of K^+^ channels over time. This can lead to an increase in intracellular K^+^ and Ca^2+^, disrupting normal cellular function and metabolism and resulting in a range of physiological and metabolic issues. Recent studies have uncovered the collaborative relationship between K^+^ channels auxiliary SUR1 and Kir6.2 gating, as well as the impact of K+ channel mutations such as KCNK11 Leu114Pro, KCNQ1Arg397Trp, KCNJ11Arg136Cys, KCNK16 Leu114Pro, and KCNMA1 Gly356Arg on diabetes mellitus and associated complications. Specifically, issues such as impaired cardiac repolarization, K_ATP_, Kir, TALK, and K_V_ channel remodeling and a higher risk of arrhythmia have been emphasized. Furthermore, structural and dysfunctional K^+^ channels (K_Ca_, K_V_ and Kir) can also affect the function of vascular endothelial and smooth muscle cells, leading to impaired vasomotor function, abnormal cell growth, and increased inflammation. These abnormalities can result in cardiovascular damage and lesions, and increase the risk of cardiovascular disease in diabetic individuals. These findings serve as a crucial foundation for a better understanding and addressing cardiovascular issues in patients with diabetes. Moreover, different drugs and treatments targeting the K^+^ channel may yield varying effects, offering promising prospects for preventing and managing diabetes and its related complications.

## Introduction

Diabetes Mellitus (DM), a complex metabolic disorder, is pathologically characterized by persistent hyperglycemia. This condition stems from insulin secretion defects (β-cell dysfunction) or insulin resistance (reduced peripheral tissue sensitivity), leading to dysregulation of glucose metabolism homeostasis [1,2]. The severity of this disease lies in its progressive multi-system damage: cardiovascular involvement accelerates atherosclerosis; 35% of end-stage renal disease cases originate from diabetic nephropathy; diabetic retinopathy remains the leading cause of blindness among working-age populations. Neuropathy contributes to severe complications like diabetic foot. These complications create a cascading effect, reducing patients’ average life expectancy by 8–12 years while driving global healthcare expenditures to grow annually at rates far exceeding those of common chronic diseases [3]. It has been reported that the global diabetic population had reached a staggering 529 million by 2021, with an overall prevalence rate of 6.1% worldwide. This alarming statistic underscores the escalating public health challenge posed by diabetes. By 2050, diabetes is projected to affect over 1.31 billion people, highlighting the urgent need for immediate action. Based on the latest WHO classification criteria, diabetes is primarily categorized into autoimmune type 1 diabetes (T1D) and metabolic type 2 diabetes (T2D), with T2D accounting for over 90% of global cases [4–6].

K^+^ channels, as critical regulators of electrophysiological activity in the cardiovascular system, play a dual pivotal role in maintaining cardiac rhythm stability and vascular function regulation. Modern studies have confirmed at these channels not only exert decisive effects in myocardial electrical activity through precise regulation of transmembrane K^+^ flow, but also participate in vascular homeostasis maintenance via multiple mechanisms [7–10]. At the myocardial electrophysiological level, these channels ensure normal cardiac pulsation through a tripartite regulatory mechanism: 1) Controlling action potential initiation by dynamically regulating cellular excitation thresholds; 2) Precisely modulating action potential duration to guarantee normal repolarization processes in cardiomyocytes; 3) Actively expelling excess intracellular K^+^ during repolarization to maintain ionic gradient balance across membranes. The K^+^ channel network in the vascular system exhibits more complex spatial distribution characteristics. In vascular smooth muscle cells (VSMCs), these channels directly influence intracellular calcium concentration through membrane potential regulation, thereby mediating the dynamic equilibrium of vasoconstriction/dilation. K^+^ channels distributed in vascular endothelial cells (ECs) indirectly regulate vascular tension by modulating endothelial-derived relaxing factor release and participate in microcirculatory permeability regulation through endothelial barrier function maintenance [11].

From a molecular biology perspective, 70 genes encoding K^+^ channels have been identified in the human genome [12]. This channel family primarily comprises: 1)Voltage-gated K^+^ channels (K_V_ channels): K_V_1 (KCNA), K_V_ 2 (KCNB), K_V_ 3 (KCNC), K_V_ 4 (KCND), K_V_ 5 (KCNF), K_V_ 6 (KCNG), K_V_ 7 (KCNQ, also named KQT), K_V_ 8 (KCNV), and K_V_ 9(KCNS), K_V_ 10 (KCNH1, also named EAG), K_V_ 11 (KCNH2, also named ERG) and K_V_ 12 (KCNH) channels. 2) Calcium-activated K^+^ channels (K_Ca_ channels): KCa1.1 (KCNMA1, BK_Ca_, slo1); KCa2.1 (KCNN1, SK1), KCa2.2 (KCNN2, SK2), KCa2.3 (KCNN3, SK_Ca_, SK3); KCa3.1 (KCNN4, IK_Ca_, SK4); and KCa4.1 (KCNT1, Slo2.2), KCa4.2 (KCNT2, Slo2.1), KCa5.1 (KCNU1, Slo3). 3) Two-pore K^+^ Channels K2P: TWIK (KCNK1), TASK (KCNK3), THIK, TRESK (KCNK18), TREK (KCNK10), and TALK (KCNK1) channels. 4) Inward-rectifying K^+^channels (Kir channels, KCNJx,): Classical Kir channels (Kir2.x); G-protein-gated Kir channels (Kir3.x); ATP-sensitive K channels (Kir6.x and auxiliary sulfonylurea receptor SUR proteins); and K^+^ transport channels (Kir1.x, Kir4.x, Kir5.x, and Kir7.x) [13]. The Table 1 summarizes the composition and functions of K^+^ channel family. The genetic diversity provides molecular foundations for specialized functional requirements in different cardiovascular tissue microenvironments, while also creating potential opportunities for targeted drug development. Several subtypes of the four types of K^+^ channels show varying degrees of alterations in diabetes. This review focuses on the latest research findings concerning modifications of K^+^ channels in diabetes.

**Table 1.** The composition and function of K^+^ channel family.

Family Name	Subtype/Alias	Gene ID	Tissue Distribution	Main Functions and Characteristics
Voltage-gated K^+^ channels (Kv channels)	Kv1.x (Shaker)	KCNA1–8	Neurons, cardiomyocyte, skeletal muscle adipose tissue; The digestive system; Genitourinary system; Immune system	Action potential repolarization, neuroexcitatory regulation, positive regulation of mitotic cell cycle G1/S transition; Positive regulation of myoblast proliferation
	Kv7.x (KCNQ, KQT)	KCNQ1–5	Heart, cochlea, neurons, germ line cells	Myocardial repolarization (Kv7.1), neuronal M current regulation; Myocardial contraction; K^+^ ion transport; Regulation of heart rate. Calmodulin dependent.
	Kv11.x (hERG)	KCNH2	cardiomyocyte, nerve cells, microglia, tumor cells	Myocardial action potential repolarization. Gene mutation can cause Long QT Syndrome Type 2 (LQT2)
Inward-rectifying K^+^ channels (Kir channels)	Kir2.x	KCNJ2,12,14	In most mammalian cells	Maintain resting membrane potential and participate in K+ homeostasis. Action potential waveform and excitability establishment of neurons and muscle tissue. Fast activation, slow deactivation
	Kir3.x (GIRK)	KCNJ3,6,9	In most mammalian cells	Neurotransmitter inhibitory regulation, heart rate control. G protein control, which plays an important role in regulating the heartbeat. Activation of beta and gamma subunits of G protein.
	Kir6.x (K_ATP_)	KCNJ8,11	Pancreatic beta cells, cardiac muscle, smooth muscle	Insulin secretion, ischemic preconditioning. G protein control and related to the sulfonylurea receptor SUR.
Calcium-activated K^+^ channels (K_Ca_ channels)	BK_Ca_ (slo1)	KCNMA1	Smooth muscle, neurons, cochlea	Vasodilation, hyperpolarization of smooth muscle cells. High conductivity, Ca^2+^ activation.
	IK_Ca_(SK4)	KCNN4	Neurons, vascular endothelial cells, T cells	Vasodilation, neuronal excitatory regulation, immune response. Calmodulin dependent, medium conductance, Ca^2+^ activation.
	SK_Ca_ (SK1–3)	KCNN1–4	Neurons, vascular endothelial cells, T cells	Vasodilation, neuronal excitatory regulation, immune response. Calmodulin dependent, intermedium conductance, Ca^2+^ activation.
Two-pore K^+^ Channels (K2P channels)	TREK (TREK1/2)	KCNK2,10	Brain, sensory neurons, cardiomyocyte	Background K^+^ current, mechanical/temperature sensitive. Control the resting film potential.
	TASK (TASK1/3)	KCNK3,9	Adrenal glands, lungs, cerebellum	Oxygen sensing, hormone secretion. pH sensitive and hypoxic regulated.
others	EAG (Kv10–12)	KCNH1,5,6	It is highly expressed in the brain and myoblasts. Expressed in tumor cells, germ cells	Cell proliferation, sperm activation. Regulates neurotransmitter release, heart rate, insulin secretion, neuronal excitability, epithelial electrolyte transport, smooth muscle contraction, and cell volume. Involved in learning and the growth of peripheral glia
	Slo2.x (Na^+^-activated)	KCNT1–2	Kidneys, neurons	Regulates neurotransmitter release, heart rate, insulin secretion, neuronal excitability, epithelial electrolyte transport, smooth muscle contraction, and cell volume. Na^+^/Cl^−^ activated.

### Expression and functional changes of K^+^ channels in diabetes

Alterations in K^+^ channels in the heart and vascular cells of diabetic patients can result in abnormal electrophysiological properties of cells, subsequently affecting the function of the cardiovascular system. Therefore, investigating these changes in these channels is crucial for understanding the mechanisms underlying diabetic cardiovascular complications, and offers new insights for developing therapeutic interventions targeting these channels. In patients with diabetes, alterations in K^+^ channels commonly manifest in several ways: 1) K^+^ channel gene mutations, which serve as a significant contributor to monogenic diabetes, leading to impaired β-cell insulin secretion and subsequent diabetes development. 2) Reduced K^+^ channel activity: The activity of K^+^ channels within cardiomyocytes and vascular smooth muscle cells of diabetic patients may be inhibited, resulting in decreased intracellular K^+^ permeability and impacting cell excitation and conduction functions. 3) Diminished density of K^+^ channels: Diabetic patients may exhibit a decrease in the density of K^+^ channels in their cardiomyocytes and muscle cells. This reduction could slow cellular repolarization, heighten heart excitability, increase the risk of arrhythmias, and disrupt vasomotor activity. 4) Alterations in K^+^ channel types: The K^+^ channel types present in the diabetic myocardium and vascular smooth muscle cells may undergo changes such as variations in channel subunit composition and abnormal channel function. These alterations can contribute to complications such as cardiovascular disease in patients with diabetes, significantly affecting their quality of life and overall health. Therefore, by targeting abnormal changes in K^+^ channels, researchers can explore tailored therapeutic approaches to help prevent and manage cardiovascular complications in diabetic individuals. In recent years, numerous studies have been conducted in this field [14–23].

**Figure 1.**
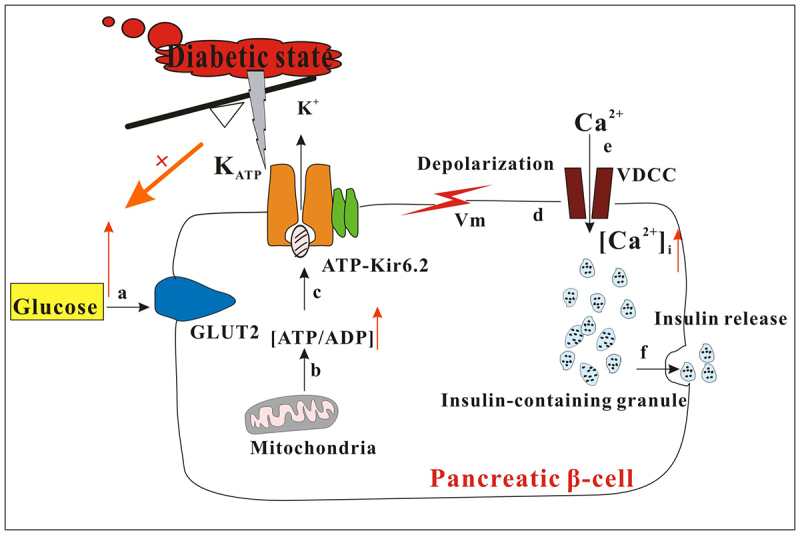
Dysregulation of insulin secretion in pancreatic β-cells under the diabetic state. One of the key mechanisms involved in insulin secretion in pancreatic β-cells is the presence of K_ATP_ channels: a. Glucose enters the β-cells through glucose transporter 2 (GLUT2). b. Glucose undergoes mitochondrial metabolism to produce ATP, leading to an increase in the intracellular ATP/ADP ratio. c. ATP binds to the Kir6.2 subunit of the K_ATP_ channel, inhibiting K_ATP_ channel activity. d. Accumulation of intracellular K^+^ ions results in membrane depolarization, activating voltage dependent calcium channels (VDCC). e. This triggers Ca^2+^ influx, leading to an increase in intracellular Ca^2+^ concentration ([Ca^2+^]_i_). f. Ca^2+^ serves as a signaling molecule to trigger the exocytosis of insulin vesicles, allowing for the release of insulin from β-cells. However, in diabetes, dysfunction of the insulin-secreting function of pancreatic β-cells or insulin resistance leads to dysregulation of K_ATP_ channel function. This disruption of the insulin secretion process (steps a-f) results in uncontrolled blood sugar levels and the occurrence of hyperglycemia.

ATP-sensitive K^+^ channels (K_ATP_ channels) are composed of four pore-forming Kir6.2 subunits and four regulatory sulfonylurea receptor (SUR) subunits. The opening of K_ATP_ channels is facilitated by PIP2 and inhibited by ATP [24]. These channels serve as a crucial link between cell metabolism and electrical activity by regulating cell membrane potential. K_ATP_ channels play a crucial role in numerous physiological processes, with a notable impact on the regulation of overall glucose levels in the body by controlling hormone secretion from islet cells. It is important to highlight that inhibition of K_ATP_ channels is essential for proper insulin secretion in response to glucose. The 2022 Banting Prize for Scientific Achievement laureates highlights the central role of K_ATP_ channels in insulin release and the metabolic regulation of this process in both health and disease. Changes in intracellular ATP and MgADP concentrations trigger the closure of K_ATP_ channels, leading to stimulation of β-cell electrical activity and insulin granule exocytosis. Mutations that activate the K_ATP_ channel gene can disrupt the channel response to ATP, resulting in diabetes. Additionally, dysregulation of K_ATP_ channels may contribute to T2D by impairing glucose metabolism and reducing glucose-induced K_ATP_ channel closure. Consequently, this leads to decreased glucose-stimulated β-cell electrical activity, ultimately resulting in reduced insulin secretion owing to impaired insulin granule exocytosis [25]. When blood sugar levels rise, glucose metabolism increases the ratio of ATP to ADP, causing closure of K_ATP_ channels. This closure leads to cell membrane depolarization, which triggers the opening of voltage-dependent calcium channels, resulting in an influx of Ca^2+^ that promotes insulin [26]. Oduori et al. [27] found that in beta cells lacking K_ATP_ channels, there is a shift in G-protein signaling from the Gs family to the Gq family. This shift explains why glucagon-like peptide-1 (GLP-1) is effective in enhancing insulin secretion in mice with K_ATP_ channel deficiencies such as diabetic KK-Ay mice. Driggers et al. [28] reported the cryoelectron microscopy structure of a K_ATP_ channel carrying the neonatal diabetes mutation Kir6.2-Gln52 Arg. This study revealed the presence of two adjacent PIP2 molecules between SUR1 and Kir6.2. Functional studies have indicated that there are two binding sites that determine channel activity. The opening of the Kir6.2 pore involves torsion of the Kir6.2 cytoplasmic domain and rotation of the N-terminal transmembrane domain of SUR1, which widens the inhibitory ATP-binding site, making it less favorable for ATP binding. The Kir6.2Gln52 Arg residue contributes to the stability of the open conformation by forming a bond with the SUR1-W51 cation–π bond. These findings highlight the cooperative relationship between SUR1 and Kir6.2 in PIP2 binding and gating, elucidate the opposing regulation of K_ATP_ channels by PIP2 and ATP, and provide insight into how Kir6.2Gln52 Arg stabilizes open channels, leading to neonatal diabetes. Figure 1 illustrates the regulation of insulin secretion by pancreatic beta cell K_ATP_ channels in patients with diabetes with high blood sugar levels.

Recent data have indicated that K_ATP_ channels play a pivotal role in glucagon secretion. In healthy individuals, a decrease in blood sugar levels triggers the release of glucagon, which in turn enhances glucose production in the liver. Unfortunately, many T1D patients do not exhibit this response, making them more prone to severe hypoglycemia. Research has demonstrated that hypoglycemia fails to stimulate glucagon secretion in islets isolated from T1D donors, and this insufficiency is also observed in non-obese T1D diabetic mice. The primary reason for this is the overproduction of somatostatin, which disrupts normal paracrine inhibition of glucagon. Typically, during hypoglycemia, the hyperpolarization of beta cells via K_ATP_ channels extends to delta cells through gap junctions, leading to suppression of action potential discharge and somatostatin release. However, when beta cells are destroyed by autoimmune reactions, this “electric brake” is lost, resulting in impaired counter-regulatory function. This phenomenon clarifies the clinical observation that some remaining beta-cell function is linked to a decreased risk of hypoglycemia [29].

In the field of research on functional alterations of diabetes-related K^+^ ion channels, mechanistic exploration in T1D remains insufficient. Current studies indicate that approximately 25% of T1D patients exhibit impaired hypoglycemia awareness, a phenomenon potentially associated with abnormal function of K_ATP_ channels composed of Kir6.2 subunits in ventromedial hypothalamic glucose-sensing neurons. Holstein et al. [30] investigated the impact of the KCNJ11 gene Glu23Lys polymorphism on impaired hypoglycemia awareness and found that while diabetes duration, C-peptide levels, and glycated hemoglobin (HbA1c) were identified as risk factors for hypoglycemia cognition impairment, this genetic polymorphism showed no significant influence regardless of adjustments for age, disease duration, or metabolic parameters. Notably, subsequent research has revealed more complex regulatory mechanisms. The Haythorne team [31] discovered that chronic exposure to the K_ATP_ channel inhibitor NN414 induces persistent conformational changes in K_ATP_ channels within hypothalamic glucose-sensing cells. These alterations may diminish channel sensitivity to endogenous metabolic factors like MgADP through interactions with SUR1 subunits. Regarding pathological mechanisms, animal studies [32] demonstrated that STZ-induced diabetic models exhibit voltage-gated ion channel remodeling in pancreatic α-cells (manifested as enhanced Na^+^ currents and weakened K^+^ currents), leading to increased action potential firing frequency and amplitude, accompanied by enlarged glucagon granules. This provides novel electrophysiological insights into the characteristic hyperglucagonemia observed in T1D. These findings suggest that K^+^ channel dysfunction may participate in T1D pathogenesis through dual central and peripheral mechanisms.

The KCNJ11 gene encodes Kir6.2, which is the primary subunit of the K_ATP_ channel expressed in tissues such as the pancreas. A specific polymorphism known as rs5219 within KCNJ11 has been identified as a risk factor for the development of T2D in humans. Gupta et al. [33] used a computational approach to predict the optimal Kir6.2. Further simulation studies were performed to assess the effects of the mutation (p.glu23lys) on both the structure and function of Kir6.2. These findings indicate that the movement of the mutated Kir6.2 residue results in K_ATP_ channel “overactivity,” leading to an “inadequate secretion” of insulin. Research has demonstrated that the most common mutation sites associated with Neonatal Diabetes Mellitus (NDM) are located within the KCNJ11 and ABCC8 genes, which encode the Kir6.2 and SUR1 subunits of the K_ATP_ channel, respectively. These genes play essential roles as subunits of the β-cell K_ATP_ channels, which are crucial components of the glucose-stimulated insulin secretion pathway. Mutations in either of these genes can disrupt insulin secretion regulation. Inactivating mutations can cause excessive insulin secretion, resulting in congenital hyperinsulinemia, whereas activating mutations can lead to diabetes [34–36]. In 2009, a 28-year-old female patient was diagnosed with diabetes and found to carry the KCNJ11Arg136 Cys mutation through whole exome sequencing, while her daughter did not have the mutation. Bioinformatics software indicated that amino acid 136 was highly conserved, and that this mutation was deleterious. KCNJ11Arg136 Cys is known to affect the structure of K_ATP_ channels, leading to changes in their function [37]. Maturity-onset diabetes of the young (MODY) comprises a diverse range of monogenic genetic disorders characterized by impaired beta-cell function. MODY can result from dysfunction of beta cell K_ATP_ channels, such as mutations in KCNJ11 (MODY13) or ABCC8 (MODY12). A previous study identified a non-synonymous coding variation in KCNK16 (NM_001135105: c.341T > C, p.Leu114Pro) that was linked to MODY. KCNK16 is the primary transcript of beta cell-specificK^+^ channels and encodes the two-pore K^+^ channel TALK-1. TALK-1 is a key regulator of β-cell electrical activity and glucose-stimulated insulin secretion. The TALK-1 Leu114Pro variant demonstrated increased functional activity compared to the wild-type, leading to altered glucose-induced membrane potential depolarization, calcium influx inhibition, and diminished endoplasmic reticulum calcium stores in mouse islets. Both mouse and human islets expressing TALK-1 Leu114Pro exhibited significantly reduced glucose-stimulated insulin secretion compared to those expressing the wild-type, suggesting that KCNK16 May be a novel pathogenic gene for MODY [38]. Furthermore, the KCNK16 Leu114Pro mutation related to MODY has been found to disrupt glucose regulation in adult mice, leading to a MODY-like phenotype and neonatal mortality by inhibiting insulin secretion in the islets during development. Nakhe developed a mutant mouse model with the KCNK16 Leu114Pro mutation. Heterozygous and homozygous KCNK16Leu114Pro mice showed higher neonatal mortality in C57BL/6J and CD-1 (ICR) genetic backgrounds, respectively. The severe hyperglycemia observed in the homozygous KCNK16 Leu114Pro neonates could be attributed to impaired glucose-stimulated insulin secretion, which can be mitigated by insulin therapy. KCNK16 Leu114Pro leads to increased whole-cell K^+^ currents in β-cells, causing reduced glucose-stimulated Ca^2+^ entry and loss of glucose-induced Ca^2+^ oscillations. Consequently, adult KCNK16 Leu114Pro mice demonstrated diminished glucose-stimulated insulin secretion and plasma insulin levels, significantly affecting glucose regulation [39]. A meta-analysis verified the association between the KCNJ11Glu23 Lys polymorphism and susceptibility to T2D [40]. Glu23 Lys is a common polymorphism in the K_ATP_ channel pore-forming subunit gene (KCNJ11). Research using a genetically engineered mouse model has shown that the Glu23 variant, when combined with a high-fat diet and obesity, hinders glucose-induced insulin secretion, and increases the risk of diabetes. In beta cells with two Glu23 risk alleles (KK), ATP inhibition of K_ATP_ channels is reduced and the threshold for glucose-stimulated insulin secretion in KK islets is increased. Therefore, in mice with the KK genotype fed a standard diet, the insulin response to glucose and blood sugar control was compromised. In the context of a high-fat diet, the KK genotype plays a more significant role, accelerating the progression of diabetes induced by diet and leading to beta cell failure [41]. Other studies have indicated that functional mutations in the Kir6 subunit of islet beta cells, which form pores in K_ATP_, are the primary cause of neonatal diabetes in humans. In insulin-secreting mouse beta cell lines, it was found that functional mutations in Kir6.1 result in a notable increase in the expression of gap junction protein 36 (Cx36), which forms gap junctions and facilitates electrical coupling between beta cells within islets. In a neonatal diabetic beta-cell model, functional mutations in Kir6.1 lead to overexpression of Cx36, exacerbating damage to glucose-stimulated Ca^2+^ oscillations [42]. Using single-nucleus analysis of transposase-accessible chromatin (snATAC-seq) and sequencing techniques, researchers have examined state-specific fasting glucose enrichment and genome-wide associations related to T2D in beta cells as well as the enrichment of other endocrine cell types. The findings revealed that a significant T2D variant in the KCNQ1 locus, rs231361, is linked to a mutually influential relationship with insulin [43]. Using bioinformatics tools and molecular dynamics simulations, Elangeeb et al. conducted a study on the Kir6.2 structure of the K_ATP_ channel. These findings revealed that mutations in Met199Arg, Arg201His, Arg206His, and Tyr330 His had a significant impact on both the structure and function of Kir6.2. These mutations have the potential to contribute to diabetes [44]. In a recent study, Zhou et al. identified a homozygous KCNQ1 mutation Arg397Trp in a patient with permanent neonatal diabetes (PNDM) without cardiovascular problems. To study the effects of this mutation, researchers introduced it into human embryonic stem cells and used CRISPR technology to create islet-like structures. Interestingly, the study found that this mutation did not affect the differentiation of pancreatic cells but did change the function of the channel. Specifically, mutated channels showed increased activity, resulting in increased early insulin secretion owing to more frequent peak events and enhanced calcium flux. However, over time, the mutated cells exhibit a decline in insulin secretion and eventually progress to a diabetic state. High glucose levels accelerate the degeneration of pancreatic cells, leading to the development of diabetes. Overall, this study sheds light on the role of KCNQ1 mutations in pancreatic function and their contribution to diabetes development [45].

K_V_ channels play a crucial role in the repolarization of various excitable tissues such as cardiomyocytes and islet beta cells. Recently, individuals with mutations in KCNQ1, encoding Kv 7.1, and KCNH2 (hERG), coding for Kv 11.1, were found to experience episodes of hyperinsulinemia and hypoglycemia after meals. These loss-of-function mutations can also lead to Long QT syndrome (LQTS), a condition that can be exacerbated by low blood sugar levels. Interestingly, patients with LQTS are more likely to have diabetes than the general population, highlighting a paradoxical link between hyperinsulinemia and heart conditions, with KCNQ1 identified as a risk gene for T2D [46]. Liang-Wang syndrome (LIWAS), a rare multi-malformation disorder caused by heterozygous mutations in the KCNMA1 gene encoding the large-conductance calcium-activated K^+^ channel (BK_Ca_ channel), was first reported in 2019. The p.gly356arg mutation in the KCNMA1 gene results in the loss of BK_Ca_ channel function and inhibits K^+^ current generation. Symptoms of this variant include developmental delays and abnormalities in the internal organs and connective tissues. Only three cases of LIWAS have been documented, one of which was associated with neonatal diabetes. One patient carrying the p. (Gly375Arg) variant of LIWAS developed diabetes within the first week of life. It is hypothesized that inactivation of the BK_Ca_ channel may affect insulin secretion by altering ion-dependent membrane activity and mitochondrial function in beta cells as well as impairing vascular reactivity in islets [47].

Research has indicated that Kcnma1 plays a crucial role in maintaining mitochondrial homeostasis, with the loss of this gene being a key contributor to skeletal muscle loss in diabetes [48]. In a study conducted by Wang et al. [49], the risk of major adverse cardiovascular events (MACE) was compared between high- and low-affinity sulfonylureas for myocardial mitochondrial ATP-sensitive K^+^ channels (mitoKATP channels). This cohort study focused on patients with T2D who initiated monotherapy with sulfonylureas between 2007 and 2016. These findings indicate that high-affinity sulfonylureas targeting the mitoK_ATP_ channel in the heart are linked to a higher risk of MACE than low-affinity sulfonylureas in the Chinese diabetic population. This study evaluated the role of the intermediate conductance Ca^2+^-activated K^+^channel (IK_Ca_) in the palmitic acid (PA)-induced migration of peripheral blood mononuclear cells (PBMCs) in 49 patients with T2D, as well as the impact of advanced glycation end products (AGEs). The results showed that 100 μM PA stimulated PBMC migration in T2D patients, and this effect was inhibited by the specific IK_Ca_ channel blocker TRAM-34. There was a positive correlation between PBMC migration in T2D patients and glycosylated hemoglobin A1c (HbA1c) levels. The expression of toll-like receptor (TLR) 2/4 and IK_Ca_ channels in PBMCs was higher in patients with elevated HbA1c levels. In PBMCs of T2Dpatients, AGEs were found to enhance PA-induced migration by upregulating TLR2/4 and IK_Ca_ channels [50]. In a study conducted by Kaya et al. the mitochondrial K^+^ channel-opening agent diazoxide was observed to mitigate STZ-indced damage to islet beta cells. Through activation of the HSP70/HSP90/TLR4/AMPK signaling pathway, diazoxide successfully protected islet beta cells from STZ toxicity [51].

There are also some related reports on the changes of K+ channels in patients with diabetes. For instance, K_ATP_ channels may play a role in the development of diabetic gastric motility disorder [52]. Impaired erectile function in diabetic db/db mice could be linked to dysfunction of BK_Ca_ channels [53]. Colburn assessed vascular K_ATP_ channel function (topical glibenclamide superfused onto fast-twitch oxidative skeletal muscle) supporting blood flow and interstitial O_2_ delivery-utilization matching (PO_2_) during twitch contractions in male, female, and ovariectomized female (F+OVX) rats. Glibenclamide reduced blood flow (convective O_2_ transport) and interstitial PO_2_ in both male and female rats but had no effect on F+OVX rats. Compared with males, females also exhibited impaired diffusive O_2_ transport, with a faster decline in interstitial PO_2_. Experimental evidence in rats demonstrates sex differences in vascular K_ATP_ channel function, supporting the initial hypothesis that oral sulfonylurea drugs may exacerbate exercise intolerance and disease incidence, especially in premenopausal women. This suggests that, in patients taking sulfonylurea drugs, there is a possibility of impaired vascular K_ATP_ channel function, leading to compromised skeletal muscle blood flow and decreased exercise tolerance. Therefore, premenopausal women taking sulfonylurea drugs may be more likely to experience adverse cardiovascular events than males. Additionally, T2D may decrease the levels of Kir6.1, the primary subunit of vascular smooth muscle in the human internal mammary artery, and alter its interaction with pinacidil [54]. In a recent study, researchers investigated the impact of dietary K^+^ intake on the overall K+ balance and kidney K^+^ processing in mice with diabetes induced by STZ. These findings revealed that a K^+^-deficient diet led to increased K^+^ loss in the urine and a decrease in the daily K^+^ balance in STZ-induced diabetic mice, resulting in hypokalemia. Conversely, STZ-induced diabetic mice exhibited an increase in the daily K^+^ balance and higher levels of plasma K^+^ when fed a K^+^-rich diet. Furthermore, abnormal levels of NaCl cotransporters, epithelial Na^+^ channels (ENaC), and extrarenal pump K^+^ channels were observed in diabetic mice fed both low and high K+ diets. Studies have shown that diabetic mice exhibit disrupted K^+^ balance and renal K^+^ processing under both low- and high-K ^+^ dietary conditions, primarily due to dysfunction in the ENaC-dependent renal K+ excretion pathway. Additionally, when mice with STZ-induced diabetes were administered high levels of dietary K^+^, SGLT2 inhibitors were found to increase urinary K^+^ excretion while decreasing plasma K^+^ levels, potentially due to heightened ENaC activity [55].

### Myocardial injury and K^+^ channel changes in diabetes mellitus

The impact of diabetes on the heart is complex and encompasses a variety of factors including cardiac electrical remodeling, increased susceptibility to arrhythmias, microvascular abnormalities, metabolic irregularities, autonomic dysfunction, and immune system dysfunction. Furthermore, even in the absence of coronary artery disease, diabetes can lead to a condition known as diabetic cardiomyopathy (DCM), which results in alterations to the function and structure of the heart muscle. Research has shown that alterations in electrical activity of the heart in individuals with diabetes are closely linked to the development of arrhythmias and sudden cardiac death. Recent studies have revealed significant changes in plasma channels for K^+^, Na^+^, and L-type Ca^2+^ currents in the hearts of patients with diabetes, as well as disruptions in calcium homeostasis and impaired systolic function. Additionally, fluctuations in insulin levels and other nutritional factors in diabetes can affect the expression of ion channels in individuals with diabetes [21].

Recent research has further confirmed the detrimental impact of chronic hyperglycemia and diabetes on cardiac electrophysiology, specifically highlighting issues such as impaired cardiac repolarization, K^+^ channel remodeling, and elevated risk of arrhythmia. Animal studies have revealed that acute hyperglycemia can lead to an increase in the inward rectifying K^+^ current (IK1) in cardiomyocytes while simultaneously causing a decrease in the amplitude and inactivation recovery time of transient outward K^+^ channels (Ito). These alterations are directly linked to the acylation of O-Glcnac within cells. Furthermore, activation of Ca^2+^/calmodulin-dependent kinase II (CaMKII) plays a significant role in these effects. By either inhibiting CaMKII activity or regulating its phosphorylation state, it may be possible to mitigate the changes occurring in the K^+^ current channels, thereby potentially reducing the incidence of cardiac complications. These findings are a crucial stepping stone toward better understanding and addressing cardiac issues in patients with diabetes [56]. Atrial fibrillation (AF) is a common condition among individuals with diabetes, particularly T2D. Recent findings suggest that T2D may be linked to specific changes in the electrical and structural characteristics of the atrium. In a study involving T2D db/db mice, researchers observed that the conduction velocity in the atria was slowed, action potential duration was prolonged, and there was uneven duplication of the left and right atrial factors closely associated with the development of AF. Furthermore, the study revealed a decrease in atrial K^+^ currents, including Ito and ultrafast delayed rectifier K^+^ currents (IKur), in db/db mice [57]. Additionally, DCM, a primary complication in individuals with diabetes that affects heart function, was investigated in a T2D mouse model. Cardiomyocytes from these mice exhibit abnormal diastolic Ca^2+^ concentrations, reduced glucose transportation, increased production of reactive oxygen species (ROS), and increased calpain activity. Moreover, cardiomyocytes showed significant damage, elevated levels of inflammatory markers such as tumor necrosis factor-α (TNF-α) and interleukin-6 (IL-6), and increased expression of nuclear factor kappa-B (NF-κB). Cell viability in mice was diminished, and there was reduced expression of the Kir6.2, SUR1, and SUR2 subunits of the K_ATP_ channel. These findings highlight the complex relationship between diabetes, atrial fibrillation, and cardiomyopathy, shedding light on the potential mechanisms underlying these conditions [58].

**Figure 2.**
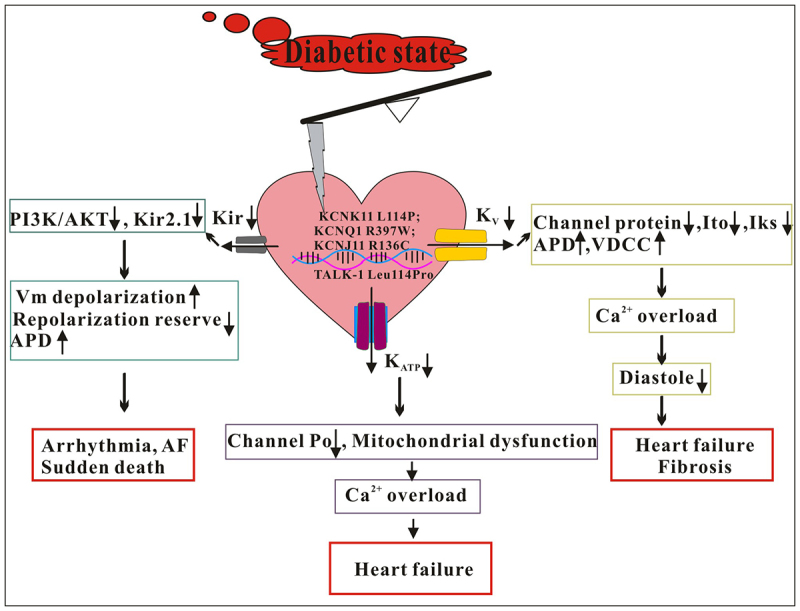
The destruction of myocardial function resulting from altered cardiomyocyte K^+^ channels under the diabetic state. Alterations in cellular K^+^ channels like K_ATP_, Kir, and K_V_ in diabetes mellitus contribute to a heightened risk of cardiovascular complications including heart failure, myocardial fibrosis, arrhythmia, atrial fibrillation, and sudden death. K_ATP_: ATP-sensitive K^+^ channels; Kir: inward rectifying K^+^ channel; K_V_: voltage dependent Ca^2+^ channel; Ito: transient outward K^+^ channel; IKs: slow-activated delayed rectifier K^+^ channel; VDCC: voltage dependent Ca^2+^ channel; APD: action potential duration; AF: atrial fibrillation.

Recent studies have examined the effect of liraglutide on myocardial electrical remodeling in diabetes mellitus. In this study, a T2D rat model was created using a high-fat diet and low-dose STZ (35 mg/kg), and various parameters, such as serum glucose, insulin, lipid levels, and modified QUICKI index, were analyzed. Additionally, the QT interval and QTc interval were measured, and the degree of myocardial interstitial and perivascular fibrosis, as well as the expression of the Ito channel α subunit and gap connexin, were observed. Furthermore, the expression of Kv 4.2/4.3 and C×43genes was assessed. The results indicated that liraglutide, metformin, and ramipril effectively inhibited diabetes-induced myocardial hypertrophy and fibrosis. Particularly, liraglutide treatment significantly improved the expression and distribution of Kv 4.2/4.3 and Cx43, leading to the prevention of diabetes-related QTc interval prolongation. This study highlights the altered expression and distribution of myocardial C×43in diabetes pathogenesis, with a potential link to the decrease in the Ito channel as the main cause of the prolonged QTc interval in T2D rats in a high-fat diet/STZ model. Overall, these findings suggest that liraglutide, akin to ramipril, plays a beneficial role in cardiac electrophysiology by regulating the expression and distribution of C×43and Ito channels in the hearts of diabetic rats [59]. KCNQ1/Kv7 is a K^+^ channel that plays a crucial role in regulating the heart rhythm and glucose levels in the body. While mutations in KCNQ1 have been linked to long QT syndrome and T2D, their impact on human pancreatic cells has been the subject of debate. Inhibition of pancreatic K_ATP_ channels is the pharmacological mechanism by which oral sulfonylurea drugs increase insulin release in patients with diabetes. However, the strength of this effect correlates with the off-target effects of sulfonylurea drugs. Currently, it is believed that this effect is related to sex differences in cardiac K_ATP_ channel function. Functional gain-of-function mutations in Kv4.3 channels can lead to a genetic disease known as Brugada syndrome (BrS), characterized by shortened cardiac action potential repolarization duration and ventricular arrhythmias. Yang, C reported a novel action of flecainide in inhibiting the Kv4.3 and Kv4.3/KChIP2 channels. The Kv4.3 and KChIP2 channels encode Ito in the heart, which is responsible for the initial phase of action potential repolarization. Flecainide has a concentration-dependent inhibitory effect on both the fast and steady-state inactivation currents of the Kv4.3 and Kv4.3/KChIP2 channels. Flecainide also accelerates the inactivation of Kv4.3 channels and shifts the steady-state activation curve in a more depolarized direction. Site-directed mutations and molecular docking studies suggest that the S301 residue of S4 and theTyr312Ala andLeu12Ala residues of the S4-S5 linker are crucial for the flecainide-mediated inhibition of Kv4.3. Furthermore, flecainide inhibits the gain-of-function Kv4.3 V392I mutation found in BrS patients in a voltage- and concentration-dependent manner. These findings indicate that flecainide inhibits Kv4.3 channels by acting on residues in the S4 and S4-S5 linker. Therefore, flecainide may have potential as a treatment for BrS [60]. In a separate study conducted by Yuan et al. the role of the KCNH2 channel in incretin secretion was investigated. By utilizing intestinal epithelial cell-specific KCNH2 knockout mice, Yuan, Y. demonstrated an improvement in glucose tolerance, accompanied by an increase in oral glucose-triggered glucagon like peptide-1 and glucose-dependent insulin-stimulating peptide secretion. Furthermore, the knockdown of KCNH2 resulted in a decrease in K^+^ current, prolongation of action potential duration, and elevation in intracellular Ca^2+^ levels, thereby facilitating the secretion of incretin from the intestines [61]. Figure 2 illustrates the increased cardiovascular risk associated with myocardial damage resulting from alterations in cardiomyocyte K^+^ channels in diabetes.

### Vascular complications and K^+^ channel changes in diabetes mellitus

In diabetics, dysfunctional K^+^ channels can impact the function of vascular ECs and VSMCs, resulting in impaired vasomotor function, abnormal cell proliferation, and heightened inflammatory response. These irregularities can cause damage and lesions in blood vessel walls, increasing the risk of microangiopathy and cardiovascular disease in affected individuals. Research has indicated that maintaining a high intracellular K^+^ concentration gradient is crucial for cellular homeostasis, with K^+^ channels serving as important regulators of K^+^ distribution across the cell membrane. In diabetes patients, persistently high blood sugar levels disrupt the balance of ion concentrations inside and outside of cells, leading to alterations in K+ channel functionality [21,62–64]: 1) Abnormal vasoconstriction: Changes in K^+^ channels can lead to abnormal vasoconstriction in diabetic patients, increasing the likelihood of vasospasm, hypertension, and other related conditions. 2) Impaired vascular endothelial function: Alterations in K^+^ channels can impair the function of vascular endothelial cells, increasing the risk of arteriosclerosis and cardiovascular disease. 3) Hemorheological abnormalities: Modifications in K^+^ channels can result in elevated blood viscosity and a higher propensity for thrombosis, leading to vascular complications, such as heart failure. 4) Difficulty in blood glucose control: Variations in K^+^ channels can affect insulin secretion and sensitivity, increasing the risk of challenges in blood glucose management and vascular complications. The activity of K^+^ channels is pivotal for regulating cell membrane potential and vascular tension. Stimulation of K^+^ channels in VSMCs results in membrane hyperpolarization and vasodilation, whereas inhibition leads to membrane depolarization and vasoconstriction. Currently, five distinct types of K^+^ channels have been discovered in VSMCs: K_Ca_, K_V_, K_ATP_, Kir, and K_2P_. When these channels do not function correctly, they can lead to vascular response issues. In cases where vascular tone is abnormal, enhancing the function and expression of K^+^ channels may provide a potential compensatory mechanism [11,17].

Research has confirmed that the vascular BK_Ca_ channel, composed of alpha subunits (BK_Ca_-α) and beta1 subunits (BK_Ca_-β1), plays a crucial role in coronary artery dilation in diabetic vessels. However, their function in these vessels is compromised. The decline in the function of the vascular BK_Ca_ channel is linked to a reduction in the expression of BK_Ca_ channel protein and alterations in its biophysical properties, a phenomenon observed in diabetic vascular diseases [11]. Additionally, changes in diabetes-related signaling pathways and transcription factors are implicated in the reduced expression of BK_Ca_ channels [62]. Sorbs2 is commonly expressed in arteries and is known for its sorbin homology (SoHo) and Src homology 3 (SH3) domains. Studies have demonstrated that Sorbs2 functions not only as a cytoskeletal protein but also as an RNA-binding protein that interacts with the BK_Ca_ channel protein and BK_Ca_-α mRNA in coronary smooth muscle cells. This interaction plays a crucial role in the regulation of the expression and function of BK_Ca_ channels. The SH3 domain of Sorbs2 is essential for its interaction with the BK_Ca_-α subunit, whereas the SH3 and SoHo domains of Sorbs2 interact with the BK_Ca_-β1 subunit. The absence of either the SH3 or SoHo domain affects the Sorbs2’s regulation of the current density of the BK_Ca_-α/BK_Ca_-β1 channel. Furthermore, Sorbs2 is a target gene of Nuclear Factor Erythroid 2-Related Factor 2 (Nrf2), with Nrf2 modulating its expression in coronary smooth muscle cells by binding to the Sorbs2 promoter. Research has also revealed that Sorbs2 knockout mice exhibit significantly reduced expression and function of BK_Ca_ channels, resulting in impaired Ca^2+^ sensitivity and coronary vasodilation. However, no changes were observed in the body weight or blood glucose levels. Interestingly, Sorbs2 expression in coronary arteries was found to be significantly decreased in mice with T2D [65]. This study examined changes in BK_Ca_ channels in human umbilical artery smooth muscle cells of patients with gestational diabetes mellitus (GDM). These findings suggest that the impairment of BK_Ca_ currents and vascular relaxation in the umbilical arteries of diabetic patients is not attributed to the dysfunction of protein kinase regulated by BK_Ca_ channels but rather to the reduced expression of BK_Ca_ channels [66]. In a separate study, the impact and mechanism of englaglizin, a sodium-glucose cotransporter 2 (SGLT2) inhibitor, on coronary artery function in patients with diabetes mellitus were investigated. A diabetic rat model was established using STZ followed by englaglizin administration. After 8 weeks, coronary artery tension was assessed and large BK_Ca_ channel currents were recorded. In addition, human coronary VSMCs were used to explore the potential mechanisms involved. The results revealed that englaglizin could reverse the glucose-induced decrease in Sirt1, Nrf2, and BK_Ca_-β1 expression, whereas the inhibition of Sirt1 with EX-527 abolished the beneficial effects of englaglizin [67]. Vascular dysfunction resulting from TD plays a significant role in the development of arteriosclerosis. Previous research has demonstrated that under conditions of hyperglycemia and diabetes, the A-kinase anchoring protein AKAP150 promotes increased vascular constriction by facilitating restructuring of the K_Ca_ channel. Zhu et al. examined the control of vascular dysfunction caused by damaged K_Ca_ channels in diabetes patients. In diabetic and AKAP150^−/−^ diabetic mice, deletion of AKAP150 reversed vascular restructuring and scarring. These findings indicated that dysfunction of the AKT/GSK3β signaling pathway resulted in reduced K_Ca_-β1 expression in the aorta of diabetic mice. Conversely, silencing of AKAP150 stimulated AKT phosphorylation and K_Ca_-β1 expression in MOVAS cells exposed to high glucose levels. Furthermore, blocking AKT activity led to reduced KCa-β1 expression, while AKAP150 siRNA treatment hindered GSK3β expression in the nucleus of high-glucose-treated MOVAS cells. In essence, the elimination of AKAP150 counteracts impaired K_Ca_ channel-mediated vascular dysfunction in diabetes via the AKT/GSK3β signaling pathway. These findings offer a new perspective and approach for managing diabetes-related vascular conditions [68]. In a study by Jiang et al. [69], the regulatory role of the IK_Ca_ channel in atherosclerotic plaques in ApoE-/- mice was explored. The research discovered that Blocking IK_Ca_ channels in vivo effectively slowed the progression of atherosclerotic lesions in diabetic ApoE^−/−^ mice fed a high-fat diet. Furthermore, in vitro experiments revealed that the levels of IK_Ca_ channels in RAW264.7 cells showed a time-dependent upregulation after treatment with high glucose combined with ox-LDL. Blocking IK_Ca_ channels also leads to a significant reduction in ox-LDL uptake by peritoneal macrophages in mice. Additional studies have indicated that IK_Ca_ siRNA and TRAM-34 (an IK_Ca_ inhibitor) decrease the expression of the clearance receptor CD36 by inhibiting the phosphorylation of STAT3. The K^+^ channel plays a crucial role in regulating vascular tone and is susceptible to the oxidative stress associated with diabetes. Understanding the impact of oxidative stress on vascular K^+^ channel function in diabetic individuals is vital for understanding vascular complications, metabolism, and cardiovascular diseases [70].

**Figure 3.**
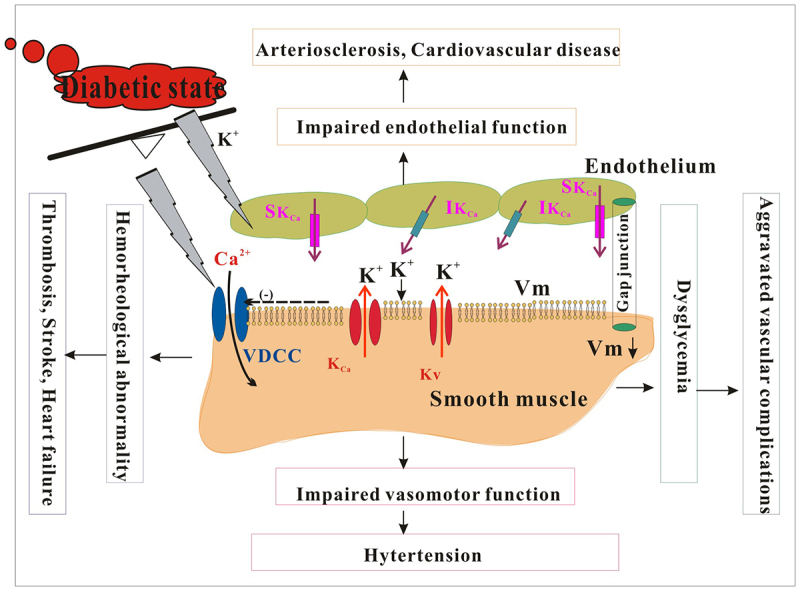
The destruction of vascular function resulting from altered vascular K^+^ channels under the diabetic state. Changes in K^+^ channels, including K_Ca_ and K_V_, in vascular smooth muscle and endothelial cells can result in various cardiovascular complications in individuals with diabetes, such as hypertension, stroke, thrombosis, atherosclerosis, and heart failure. K_Ca_: Ca^2+^ activated K^+^ channel; IK_Ca_: intermedia conductance Ca^2+^ activated K^+^ channel; SK_Ca_: small conductance Ca^2+^ activated K^+^ channel; K_V_: voltage-gated K^+^ channels; VDCC: voltage dependent Ca^2+^ channel; vm: membrane potential.

The hypothesis that SKA-31, a K_Ca_ channel activator, can restore endothelium-dependent vasodilation in T2D rats has been confirmed [71]. SKA-31 has been utilized in the acute treatment of isolated resistance arteries in both T2D rats and humans, with results demonstrating a significant improvement in endothelium-dependent vasodilation. Male Sprague Dawley (SD) and T2D Goto-Kakizaki (GK) rats were injected intraperitoneally with 10 mg/kg SKA-31. Reactive congestion/flow-mediated dilation (FMD) of the femoral artery after the release of the occlusive cuff in the distal posterior limb and changes in the diameter of the left main coronary artery following isoflurane inhalation were monitored. The findings indicated that T2D GK rats exhibited a weak FMD response, but prior administration of SKA-31 restored FMD levels comparable to those of control SD rats. In T2D GK rats, inhaling of 5% isoflurane alone did not result in an increase in coronary artery diameter; however, a strong vasodilation response was observed after treatment with SKA-31. This study suggests that enhancing in vivo K_Ca_ channel activity can effectively restore endothelium-dependent vasodilation in T2D rats with peripheral endothelial dysfunction. Marinko conducted a study on the impact of H_2_S on the K^+^ channel subtype in the vascular relaxation mechanism of an isolated human mammary internal artery (HIMA). The study revealed that H_2_S caused relaxation in phenylephrine-induced contractions of HIMA in a concentration-dependent manner. Various K^+^ channel blockers, such as iberiotoxin, glibenclamide, 4-aminopyridine (4-AP), and margatoxin, were found to significantly inhibit this relaxation. However, the combination of apamin/TRAM-34 weakened the degree of HIMA relaxation. Inhibitors of the NO pathway, including L-NAME, KT5823, and the cyclooxygenase inhibitor indomethacin, counteracted the effects of H2S. When the synthesis and release of NO/prostaxinin were blocked, the combination of apamin/TRAM-34 and glibenclamide further decreased the H2S-induced vasodilation. Additionally, nifedipine partially mitigated the H2S-induced relaxation of HIMA. These results suggest that H_2_S can lead to concentration-dependent relaxation of isolated HIMA by potentially opening various K^+^ channel subtypes, including K_ATP_, BK_Ca_, IK_Ca_, SK_Ca_, and K_V_ and K_V_1.3. Furthermore, this relaxation mechanism involves activation of the NO pathway and interference with extracellular Ca^2+^ influx [72]. Mahmood conducted a study on the interaction between melatonin (MEL) and K+ channels in the vasodilation of aortic ring angiotensin 1–7 (Ang 1–7) in diabetic male albino rats. A study found that MEL enhances the vasodilator effect of Ang 1–7 through its receptor and antioxidant activity, indicating its potential in combating oxidative stress and diabetes-related diseases [73]. In another study, the vasodilatory effects of trigliptin, a dipeptidyl peptidase-4 inhibitor, and its related mechanisms were investigated. Trigliptin induces vasodilation in a dose-dependent manner. Pretreatment with the K_ATP_ channel inhibitor glibenclamide, BK_Ca_ channel inhibitor paxilline, and Kir channel inhibitor Ba^2+^ did not affect the vasodilation caused by trigliptin. However, pre-treatment with the Kv channel inhibitors 4-AP and tetraethylammonium significantly reduced the vasodilation induced by trigliptin, indicating that vasodilation is linked to Kv channel activation. Additionally, inhibitors of the muscle/endoplasmic reticulum Ca^2+^ -ATPase (SERCA) pump significantly dampened the vasodilatory effects of trigliptin. These findings suggest that the vasodilator effect of trigliptin is associated with the activation of K_V_ channels (mainly the K_V_ 7.X subtype) and the SERCA pump. Therefore, caution should be exercised in patients with hypotension and diabetes when using trigliptin [74,75]. Jung et al. investigated the vasodilatory effects of alogliptin, an oral hypoglycemic agent belonging to the dipeptidyl peptidase-4 (DPP-4) inhibitor class, on aortic rings induced by phenylephrine (Phe). These results indicated that alogliptin exerted a dose-dependent vasodilation effect. Interestingly, the vasodilatory effect of alogliptin was significantly attenuated by pretreatment with the K_V_ channel inhibitor 4-AP, while treatments with the internal rectifier K^+^ (Kir) channel inhibitor Ba^2+^, K_ATP_ channel inhibitor glibenclamide, and BK_Ca_ channel inhibitor paxilline had no impact on the vasodilatory response. Furthermore, pretreatment with the SERCA pump inhibitors thapsigargin and cyclopyrazinic acid effectively dampened the vasodilatory effect of alogliptin, and inhibitors of guanylate cyclase (ODQ), PKG (KT 5823), adenylate cyclase (SQ 22,536), and PKA (KT 5720) did not alter the vasodilation induced by alogliptin. Additionally, removal of endothelial cells or pretreatment with nitric oxide (NO) synthase inhibitors L-NAME and SK_Ca_, and IK_Ca_ channel inhibitors apamin and TRAM34 did not affect the vasodilatory effects of alogliptin. These findings suggest that the mechanism underlying alogliptin-induced aortic smooth muscle relaxation in rabbits may involve the activation of K_V_ channels and SERCA pumps, independent of other K^+^ channels, cGMP/PKG or cAMP/PKA signaling pathways, and endothelial cell participation [76]. Figure 3 illustrates vascular complications resulting from alterations in K^+^ channel in diabetics.

## Conclusions

This review summarizes two decades of research progress on K^+^ channel pathophysiological alterations in individuals with diabetes mellitus, while systematically elucidating emerging evidence from the most recent five-year period that highlights the critical involvement of these ion channels in the pathogenesis of diabetic cardiomyopathy and associated micro/macrovascular complications. Given the significant global impact of diabetes on cardiovascular health, it is imperative to understand the alterations occurring in K^+^ channels within the cardiac and vascular cells of individuals with diabetes. Various abnormalities in the K^+^ channels have been observed in patients with diabetes, including gene mutations, type variations, reduced activity, and diminished density. These changes not only affect cardiac electrophysiology, but also elevate the risk of impaired cardiac repolarization, K^+^ channel remodeling, and arrhythmias. Furthermore, diabetic hyperglycemia and oxidative stress can adversely affect the key role of these channels as regulators of vascular tone. Consequently, modulating the activity and function of these channels are promising approaches for preventing and managing cardiovascular complications associated with diabetes. Future investigations should delve deeper into the regulatory mechanisms and pivotal targets of K^+^ channels, explore the interplay between insulin resistance and these channels, delineate the specificity and functional diversity of different channel subtypes, develop targeted therapeutic agents, and assess the potential of gene-specific interventions to restore K^+^ channel function and reverse β-cell dysfunction. This field faces challenges in drug development owing to the complexity and safety concerns involved in targeting these channels. Understanding alterations in K^+^ channels is paramount for elucidating the pathophysiology of cardiovascular complications in diabetes and for devising innovative therapeutic strategies that target these channels.

## Data Availability

Data sharing is not applicable to this article as no new data were created or analyzed in this study.
